# Word Detection in Sung and Spoken Sentences in Children With Typical Language Development or With Specific Language Impairment

**DOI:** 10.5709/acp-0177-8

**Published:** 2015-12-31

**Authors:** Clément Planchou, Sylvain Clément, Renée Béland, Nia Cason, Jacques Motte, Séverine Samson

**Affiliations:** 1 Neuropsychology: Audition, Cognition, Action, PSITEC Laboratory (EA 4072), Department of Psychology, University of Lille, France; 2 Pediatric Neurology Unit, American Memorial Hospital, University Hospital of Reims, France; 3 Department of Speech Therapy and Audiology, University of Montreal, Canada; 4 Research Centre in Neuropsychology and Cognition (CERNEC ), University of Montreal, Canada

**Keywords:** word detection, sung sentences, language development, Specific Language Impairment

## Abstract

Background: Previous studies have reported that children score better in language tasks using sung rather than spoken stimuli. We examined word detection ease in sung and spoken sentences that were equated for phoneme duration and pitch variations in children aged 7 to 12 years with typical language development (TLD) as well as in children with specific language impairment (SLI ), and hypothesized that the facilitation effect would vary with language abilities. Method: In Experiment 1, 69 children with TLD (7–10 years old) detected words in sentences that were spoken, sung on pitches extracted from speech, and sung on original scores. In Experiment 2, we added a natural speech rate condition and tested 68 children with TLD (7–12 years old). In Experiment 3, 16 children with SLI and 16 age-matched children with TLD were tested in all four conditions. Results: In both TLD groups, older children scored better than the younger ones. The matched TLD group scored higher than the SLI group who scored at the level of the younger children with TLD . None of the experiments showed a facilitation effect of sung over spoken stimuli. Conclusions: Word detection abilities improved with age in both TLD and SLI groups. Our findings are compatible with the hypothesis of delayed language abilities in children with SLI , and are discussed in light of the role of durational prosodic cues in words detection.

## Introduction

Language and music perception abilities develop in early infancy. A number of studies have suggested that these two cognitive functions are subserved by common cerebral structures (Koelsch, Gunter, Wittfoth, & Sammler, 2005; Patel, 2003; Patel, Gibson, Ratner, Besson, & Holcomb, 1998) and, thus, could influence each other during their acquisition. Research in the development of speech perception indicates that syllable perception develops in the first days of life. For instance, babies aged four-days are able to discriminate disyllabic and trisyllabic stimuli matched for acoustic duration (Bijeljac-Babic, Bertoncini, & Mehler, 1993; Christophe, Pallier, Bertoncini, & Mehler, 1991). At about the age of four years, French-speaking children are able to segment speech into syllables, and at the age of six years, children are able to detect syllables in sentences (Blaye & Lemaire, 2007).

This ability can, however, be impaired in neurodevelopmental disorders. For instance, it has been reported that children with Specific Language Impairment (SLI) present deficits in syllable perception. SLI, also known as *developmental dysphasia* (e.g., Benton, 1964), is a heritable neurodevelopmental disorder—that is diagnosed when a child has difficulties learning to produce and/or understand speech for no apparent reason (Bishop, Hardiman, & Barry, 2012; Bishop & Norbury, 2008). On comparing a group of 8-year-olds with SLI with two groups of children with typical language development (TLD), one composed of 6-year-olds and the other one composed of 8-year-olds, Montgomery (2002) found that children with SLI were slower to detect monosyllabic words in spoken sentences. In this test, children had to detect monosyllabic words presented in pictures followed by auditory stimuli in a set of 84 spoken sentences. They were instructed to press a button as quickly as possible when they heard the monosyllabic target word. No significant difference in the mean number of correct detections was found between the three groups, but in the TLD group, the older (8-year-old) children were faster than the younger children, and the younger (6-year-old) children were faster than the 8-year-old children with SLI. By contrast, this group effect was not found in a tone detection task involving speed and accuracy in detecting a 2000 Hz tone. Based on these findings, the authors drew three conclusions: a) the age effect in TLD is linked to the development of language skills, b) word detection in children improves from 6 to 8 years, and c) children with SLI encounter difficulties in detecting words in sentences.

In previous work, Montgomery, Scudder, and Moore (1990) tested the influence of linguistic context on word recognition in 8-year-olds with SLI and in a group of children with TLD matched for language level (*M*_age_ = 6 years). The stimulus set consisted of pairs of spoken sentences. The first sentence provided the linguistic context while the second contained the monosyllabic target word, whose location in the sentence varied among the 3rd, 7th, and 10th syllable position. The children had to detect the target that was presented in the picture and auditory stimulus as quickly as possible. The results revealed a significant position effect for mean reaction times (RTs) in both groups, who detected targets in the 7th and 10th syllable position more quickly than those in the 3rd. The authors suggested that both groups of children were influenced by the meaning of the sentence when detecting the monosyllabic target word, because they were faster after having heard more words of the sentences. The effect of linguistic context on word detection was further investigated in older children. Montgomery (2000) tested word detection in 12 children with SLI (*M*_age_ = 9 years) and two groups of children with TLD: one age-matched group and one group matched for receptive syntax level (*M*_age_ = 7 years). The procedure was similar to the one used in previous studies. The results showed an age effect on word detection in the children with TLD, whereby the older TLD group (age-matched group, *M*_age_ = 9 years) was faster than the younger TLD group (receptive syntax level-matched group, *M*_age_ = 7 years). Moreover, the children in the younger TLD group, although matched for receptive syntax level, were faster in word detection than the children with SLI. The results also showed a significant position effect in all three groups, with shorter RTs found for monosyllabic word targets located in the middle of the sentence than at the beginning. This finding confirmed that the two age groups of children with TLD, and the group of children with SLI, used the meaning of the sentence to aid detection of the monosyllabic target words.

The above studies have shown that children with SLI may have impaired abilities in auditory detection for verbal units. As the present study also aims to investigate the ways in which the perceptual abilities in language and music could possibly interact, an important source of information comes from studies that have compared auditory processing of spoken and sung stimuli.

In adults, Schön et al. (2008) have reported an improved auditory memory for sung over spoken stimuli. In their study, participants studied six trisyllabic nonword stimuli that were either spoken or sung. Results revealed that participants better recognized sung than spoken nonwords, which suggests that pitch variations facilitate word segmentation. In babies aged 6 to 8 months, Thiessen and Saffran (2009) used a head-turn preference procedure with 12 series of five heard digits that were either spoken or sung. Results in the recognition phase showed that the infants stared longer at lights when they were listening to a new rather than to an old sequence of numbers, and that this difference in duration was larger in the sung than in the spoken condition. The authors concluded that if infants are more sensitive to changes in the sung than in the spoken stimuli, singing would likely facilitate their verbal learning. Using the same head turn preference procedure, Lebedeva and Kuhl (2010) tested 11 month-old babies’ detection of differences in syllable order within quadrisyllabic nonwords that were either sung or spoken. In both spoken and sung stimuli, in a proportion of trials, the order of the last three syllables was modified while keeping the melody intact in the sung condition. Results confirmed those of the previous study in that looking times were longer for novel versus familiar quadrisyllabic nonwords, and that the difference in looking time was greater when stimuli were presented in the sung compared to the spoken condition. The advantage for sung over spoken speech input appears very early in language acquisition and persists into adulthood (Schön et al., 2008).

Much of the evidence above has shown that the detection of monosyllabic target words was faster when targets were located at the end than at the beginning of sentences. The authors of these studies have suggested that the children were influenced by linguistic context—that is, processing of the meaning of the sentence helped them to detect the monosyllabic target word. Another manipulation of context for sung stimuli involves modifying the harmonic structure of sung stimuli according to the standards of Western music. This is exactly what was done in a series of experiments conducted with French-speaking adults (Bigand, Tillmann, Poulin, D’Adamo, & Madurell, 2001) and French-speaking children (Schellenberg, Bigand, Poulin-Charronnat, Garnier, & Stevens, 2005). In Schellenberg et al.’s study, children aged 6 to 11 years were presented with eight-chord sequences. The chords were composed of four notes, each played by a voice synthesizer and corresponding to one syllable. The chord sequences were composed of different syllables in French, and the target syllable, which was always in the last position of the chord-sequence, was either /*di*/ or /*du*/. The participants were required to decide whether the target was sung on a syllable containing the phoneme /*i*/ or /*u*/. The musical context was manipulated so that the target chord acted as either a tonic chord, or a subdominant chord. Schellenberg et al. hypothesized that if the participants were influenced by the musical context, their detection of the target vowel /*i*/ or /*u*/ should be faster in syllables corresponding to the tonic chord because, although both chords are congruent in that context, the tonic is more expected than the subdominant chord in Western music. Results confirmed this hypothesis by showing that phoneme detection was faster in tonic chords than in subdominant chords. These findings indicated that school-aged children showed implicit knowledge of syntactical rules characterizing the Western musical system, and that phoneme detection was influenced by harmonic context.

In Western music, sung syllables are longer than spoken syllables (Scotto di Carlo, 2007). The pitch variations in songs generally correspond to a tonal melody, whereas pitch variations in speech do not. Hence, sung syllables may be detected more quickly due to their longer duration (Kilgour, Jakobson, & Cuddy, 2000) because pitch variations follow a tonal melody, or a combination of these factors. In the present study, we manipulated these variables independently to assess their contribution to the development of monosyllabic word detection abilities in French-speaking children with TLD, and those with SLI. In Experiment 1, four groups of school-aged children detected words in three conditions: a) sentences spoken at a slow rate of speech (Slow speech condition), b) sentences sung on pitches extracted from the spoken sentences (Prosody condition), and c) sentences sung on pitches from the original score (Sung condition). In order to test the effect of pitch variations independently from syllable length, the durations of the phonemes were equalized across the three conditions. In Experiment 2, we tested two groups of school-aged children in the same three conditions, and added a fourth testing condition in which sentences were naturally spoken (Normal speech condition)—that is, speech without any acoustic modification. Finally, in Experiment 3, we tested children with SLI and their matched controls in the four conditions.

To summarize, the ability to segment speech into syllables emerges early in infancy, particularly in the French language (Mehler, Dommergues, Frauenfelder, & Segui, 1981). Monosyllabic word detection ability improves between the ages of 6 to 10 years in children with TLD and is facilitated when it occurs later rather than earlier in a sentence. Speech processing also appears to be facilitated in both babies and adults when syllables are sung rather than spoken. Moreover, the phoneme detection in sung syllables is faster when the target corresponds to the harmonic expectations than when it does not. Together, these findings indicate that speech perception is influenced by the verbal context, as well as by musical context when stimuli are sung. To our knowledge, no studies have investigated the detection of monosyllabic words in sung versus spoken sentences in school-aged children with TLD or with SLI, who experience difficulties detecting words in sentences. In three experiments, we were able to assess the effects of spoken versus sung speech on word detection, as well as the impact of linguistic context, which was assessed by varying the position of the target words in the sentences. These three experiments allowed us to investigate the strength of this effect as a function of the child’s level of language development. Examining the impact of verbal and musical context on language processing by children with SLI is potentially important, not only because it can reveal further insights into their language development profile, but also because it can assess the potential for music to be used as a tool for speech therapy treatment.

## EXPERIMENT 1: EFFECT OF PITCH VARIATIONS ON WORD DETECTION

This experiment aimed to assess the effect of pitch variations on RTs in a word detection task in children aged 7 to 10 years. Three types of sentences were created: 1) sentences from nursery rhymes sung to the tune of the original melodies (Sung condition), 2) sentences sung using the pitch variations extracted from a normal speech rate (Prosody condition), and 3) sentences spoken using the duration of the syllables in the Sung condition (Slow speech condition).

According to the literature, abilities in word detection improve with language development, and, thus, word detection should get quicker as children get older. Children should also be quicker when the words are sung (Sung and Prosody conditions) than when they are spoken (Slow Speech condition). Following Montgomery (2000, 2002; Montgomery et al., 1990), if linguistic context facilitates word detection, children should be quicker to detect word targets when they are located at the end rather than at the beginning of sentences. Following Bigand et al. (2001) and Schellenberg et al. (2005), if the children are influenced by the harmonic context, they should detect words faster when the melodic context is expected (Sung condition) rather than unexpected (Prosody condition). Moreover, if the linguistic context and the melodic context have additive effects, the difference in RTs for targets located at the beginning versus at the end of sentences should be larger in the Sung than in the Prosody conditions.

### Method

#### Participants

Sixty-nine children (39 girls and 30 boys) aged 7 to 10 years were recruited in three different schools in Reims and in Lille (France) to participate in this study. They were subdivided into four age groups: 7 years (*n* = 15, *M*_age_ = 7.5 years), 8 years (*n* = 19, *M*_age_ = 8.4 years), 9 years (*n* = 21, *M*_age_ = 9.5 years), and 10 years (*n* = 14, *M*_age_ = 10.7 years). All participants were native French-speaking children attending regular school, and none of them had a documented history of language problems or neurological disorders according to a questionnaire completed by their parents.

#### Stimuli

*Sentences*. Twenty-four sentences containing 12 to 20 syllables were extracted from a repertoire of songs for children (Appendix A). Very popular songs (e.g., *Frčre Jacques* [Brother John]), songs containing lyrics with word repetition, slang words, incorrect syntax, or dialectal words were excluded. The 24 selected sentences were each produced by a professional singer in the three conditions, resulting in a total of 72 sentences. In all three conditions, the singer was instructed to pronounce one syllable per pulsation.

In the Sung condition, each sentence was sung using the original score. In the Slow speech condition, each sentence was produced slowly, respecting the number of syllables produced in the Sung condition (one syllable per note and per pulsation). In the Prosody condition, we first identified the pitch of each syllable in the Slow speech condition using the Melodyne software (Neubaecker, Gehle, Kreutzkamp, & Granzow, 2008) and adjusted each pitch to the closest note value to create a new score. The professional singer was then instructed to sing the sentence using that score. The Prosody condition was thus composed of syllables sung on musical sequences that do not respect the rules of harmony in Western music (see the example in Appendix B).

**Figure 1.**
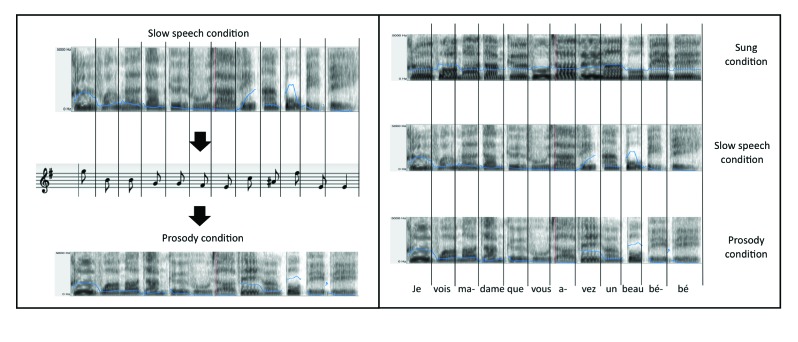
Experiments 1, 2, and 3. Left: Example of a sentence’s transformation from the Slow speech condition to the Prosody condition. The extracted pitch contours were determined by finding the closest musical pitch to the mean pitch for each syllable in the Slow speech condition. Right: Example of the three conditions with the sentence “Je vois madame que vous avez un beau bébé”, whereby syllable durations are equal across conditions (illustrated by vertical lines). Solid lines represent the F0.

As mentioned earlier, because we were interested in testing the independent effect of pitch variations we edited the acoustic signal to equalize the duration of syllables across the three conditions. In order to do that, we first measured the duration of each phoneme of the 24 sentences in the Sung Condition. Using Praat Software (Boersma & Weenink, 2009), we edited the acoustic signal of the Slow speech and Prosody conditions by either lengthening or shortening the duration of each phoneme to make it equal to the duration of each phoneme in each syllable in the Sung condition. Figure 1 illustrates an example of a sentence “Je vois madame que vous avez un beau bébé” [I see Madam that you have a nice baby], with equal syllable durations in all three conditions. Duration of the sentences varied from 4,983 ms to 12,758 ms, with a mean of 7,920 ms (*SD* = 1,553 ms).

*Target words*. Twenty-four monosyllabic words (14 Consonant-Vowel patterns CV; 4 CVC; 3 CCV; 2 CVV; 1 CCVC), were selected as targets. One word was used in each sentence. Half of the targets occurred in the first half of the sentence and half in the second part of the sentence, which from now on are referred to as Beginning position, and End position, respectively. To facilitate the task and minimize the number of trials in which children missed the target, the first three syllables and sentence final syllables were never selected as targets. The mean duration of the 24 target words was 476 ms (*SD* = 86 ms) and ranged from 357 ms to 662 ms.

*Fundamental frequency (F0) of syllables in the three conditions*. The analysis of pitch variations within each condition revealed that the mean fundamental frequency (F0) of syllables in the Sung condition was higher (*M*_F0_ = 202 Hz) than in the Slow speech condition (*M*_F0_ = 136 Hz), *t*(46) = 8.8, *p* < .001, and the Prosody condition (*M*_F0_ = 151 Hz), *t*(46) = 4.8, *p* < .001, the last two conditions not being different, *t*(46) = 1.8, *ns*. Within each condition, each F0 value of each syllable was converted into semitones in order to assess the difference in semitones between successive syllables. The mean semitone difference between successive syllables was smaller in the Sung condition (mean difference in absolute value, *MD* = 2.1 semitones) compared to the Slow speech condition (*MD* = 4 semitones), *t*(46) = 5.7, *p* < .001, and Prosody condition (*MD* = 3.9 semitones), *t*(46) = 5.6, *p* < .001, and as expected, the mean difference between the last two conditions was not significant, *t*(46) = 0.3, *ns*. To verify that the pitch saliency of the target words was comparable across conditions, we calculated the mean difference between the pitch of the target word and the pitch of the preceding syllable. This mean difference in the Sung condition (*MD* = 2.7 semitones) did not differ significantly from the mean of the Slow speech (*MD* = 3.4 semitones), *t*(46) = 1, *ns*, or Prosody condition (MD = 3.5 semitones), *t*(46) = 1.1, *ns*. The mean duration of the target words, minimum and maximum F0 in the sentences, mean F0 of the sentences, and mean F0 of the target words in the three conditions are reported in Table 1.

**Table 1.** Sentence Stimuli Characteristics for Experiments 1, 2, and 3. Mean Duration and Mean F0 of the Sentences, Target Words, and Non-Target Words in the Three Conditions

	Sentences		Target Syllables		Non Target Syllables	
	Mean F0(min-max)	Mean Duration(min-max)	Mean F0(min-max)	Mean Duration(min-max)	Mean F0(min-max)	Mean Duration(min-max)
Sung Condition	202 Hz(98 Hz - 349 Hz)	7920 ms(4983 ms - 12758 ms)	202 Hz(123 Hz - 292 Hz)	494 ms(349 ms - 714 ms)	200 Hz(98 Hz - 349 Hz)	488 ms(247 ms - 920 ms)
Prosody Condition	151 Hz(75 Hz - 415 Hz)		145 Hz(82 Hz - 247 Hz)		149 Hz(75 Hz - 415 Hz)	
Slow speech Condition	136 Hz(78 Hz - 247 Hz)		131 Hz(87 Hz - 233 Hz)		135 Hz(78 Hz - 247 Hz)	

#### Procedure

Each participant wearing headphones (Sennheiser HD 265) was seated in a quiet room in front of a computer (Dell-Latitude 531). To engage children, the word detection task was presented as a video game. Each child was instructed that s/he had to help an animated character understand what its new friend was telling it. The target words were spoken (not sung) by a male speaker, different from the speaker who recorded the sentence stimuli. One thousand five hundred milliseconds after hearing the word, a sentence was presented. The child was instructed to press the space bar as quickly as possible on hearing the target word. The sentence was stopped as soon as the child pressed the space bar. If the child did not respond, the whole sentence was presented. The next trial started immediately thereafter. RTs were recorded using E-Prime 2 (Psychology Software Tools).

Twenty-four sentences were separated into three sets of 8 sentences. Each child was tested on 48 trials, which were divided into three blocks of 16 sentences. Each child heard one sentence in two of the three conditions in order to limit fatigue and learning effects (see Appendix C and D for the composition of the blocks). Sentences and conditions within a block were presented in a random order. The 16 blocks were separated by a pause of a variable duration (1 to 3 min) determined individually by the participant, resulting in a total testing duration of around 15 min.

### Results

#### Data analysis

In order to determine whether a response was valid or not, we had to verify that the child did not press the space bar before the target word occurred in the sentence. Responses were categorized as valid if, and only if, the RT measured at the beginning of the target was not shorter than 150 ms, and not longer than 1,500 ms. The lower limit of 150 ms was used because it corresponds to the shortest RT recorded in a detection task for simple auditory stimuli (Montgomery & Leonard, 1998). The upper limit, 1,500 ms, was chosen because, keeping in mind that no targets corresponded to the sentence final syllable, it corresponds to the smallest duration between the end of the target and the end of the sentence. Using these criteria, the mean rate of valid responses as a function of the total number of target words was 77% across all the participants. Among the 615 invalid responses, 320 (52%) corresponded to responses given before the target was heard, whereas 188 (31%) corresponded to RTs longer than 1,500 ms. The remaining 107 (17%) invalid responses corresponded to responses with an RT between 0 and 150 ms.

**Figure 2.**
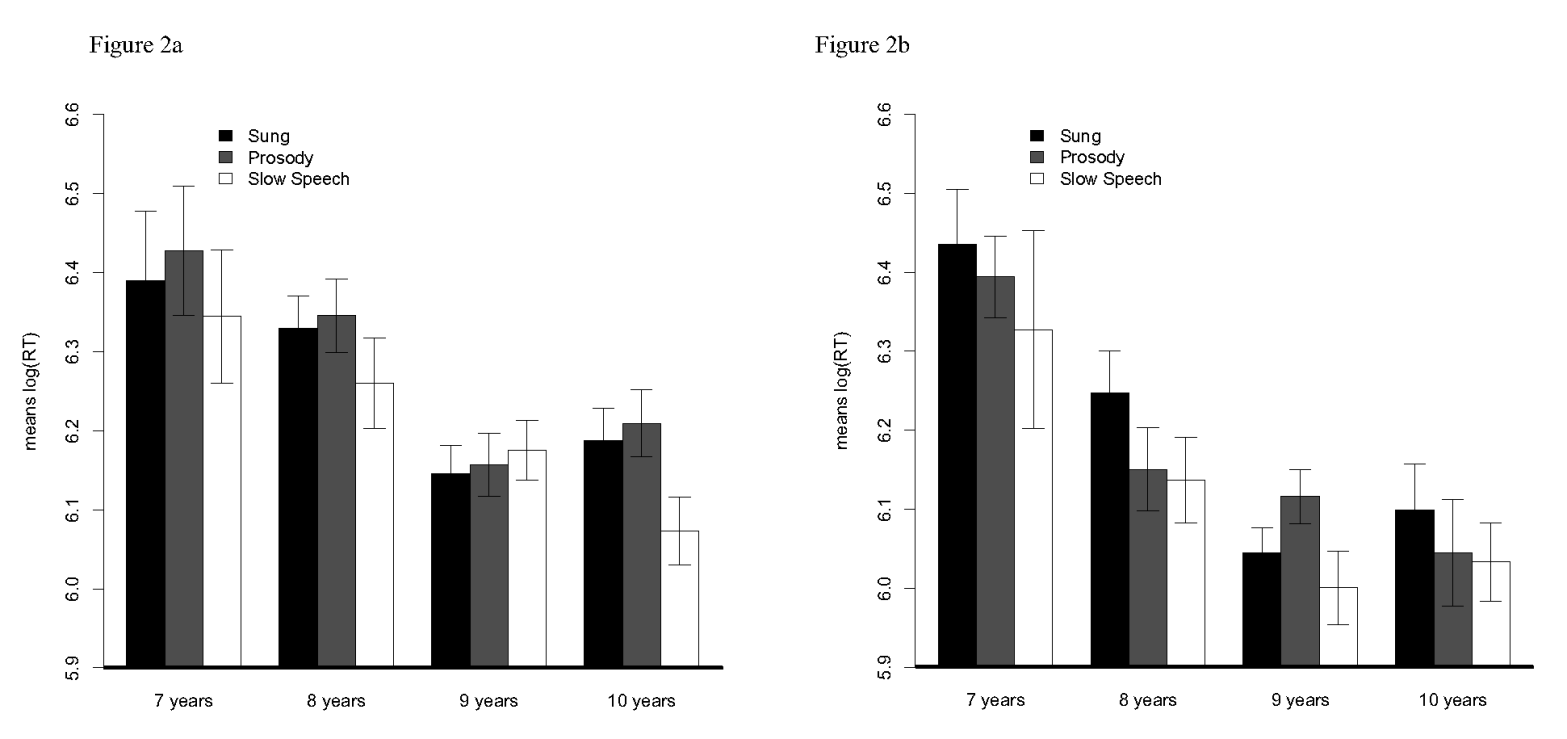
Mean logarithmic reaction time [log(RT)] in Sung (black bars), Prosody (grey bars) and Slow Speech (white bars) conditions for children aged 7, 8, 9, and 10 years for a. targets located at the beginning position and, b. targets located at the end position of Experiment 1. Error bars represent standard errors of mean.

The analysis of RTs was run only on those participants and sentences for which the valid response rate was higher than 70%. The application of this criterion led to the exclusion of all the data from 17 participants and all the data from five sentences. Mean RT and standard deviations were then calculated on kept data. RTs deviating from the mean by two or more standard deviations were removed from the data set. A logarithmic transformation (ln) was applied to the RTs to normalize the data. An analysis of variance (ANOVA) with repeated measures was run using the logarithm of RT values as the dependent variable. The ANOVA had two between-subjects factors: Age, with four levels (7 years, 8 years, 9 years, 10 years), and Target Position, with two levels (Beginning, End), and one within-subject factor Condition, with three levels (Slow speech, Sung, Prosody). The results, illustrated in Figure 2, revealed a main effect of Age, *F*(3, 48) = 11.84, *p* < .001. Post-hoc comparisons with Holm-Bonferroni corrections showed that the 8-year olds (*M*_log(RT)_ = 6.24) responded significantly faster than the 7-year olds (*M*_log(RT)_ = 6.39), and the 9-year olds (*M*_log(RT)_ = 6.11) responded significantly faster than the 8-year olds, but that the 10-year olds (*M*_log(RT)_ = 6.11) did not respond faster than the 9-year olds. The ANOVA also revealed a significant effect of Condition, *F*(2, 96) = 3.89, *p* < .03, with shorter RTs in the Slow speech condition (*M*_log(RT)_ = 6.14) than in both the Prosody (*M*_log(RT)_ = 6.20) and the Sung condition (*M*_log(RT)_ = 6.20). Post-hoc comparisons revealed that the Slow speech condition differed significantly from both the Prosody and Sung conditions. The ANOVA revealed no significant interactions between Age and Condition factors, *F*(6, 96) = 0.54, *ns*. A significant effect of Position was found, *F*(1, 48) = 31.02, *p* < .001, with shorter RTs when the target was located at the end (*M*_log(RT)_ = 6.13) than when it was located at the beginning (*M*_log(RT)_ = 6.23), and this was irrespective of the Condition. The Position by Condition interaction was not significant, *F*(2, 96) = 0.70, *ns*, neither was the Age by Condition interaction, *F*(6, 96) = 0.54, *ns*, and finally, the triple Age by Target Position by Condition interaction was not significant, *F*(6, 96) = 1.75, *ns*. Mean RTs in the three conditions as a function of Age are reported in Figure 2a for word targets located at the beginning of sentences, and in Figure 2b for word targets located at the end of sentences.

### Discussion

The first result of this experiment was that word detection in sung or slowly spoken sentences was difficult for 7- to 10-year olds. The percentage of valid responses was much lower than expected, leading us to exclude a large part of the responses (49.5%). The percentage of correct responses in the present experiment was also lower than in comparable studies by Montgomery (2000; Montgomery et al., 1990; Montgomery & Leonard, 1998) in English-speaking children, wherein the authors reported mean correct response rates between 88% and 98%. A number of factors may have contributed to the greater level of difficulty of the word detection task in the present study. Firstly, whereas we used a mix of sung and spoken sentences, Montgomery used spoken sentences only. Secondly, the children determined the duration of the pause between conditions and some of them may have overestimated their detection abilities, so that they could have benefited from a longer pause.

Notwithstanding the high rate of invalid responses, the task seemed easier for older children, since RTs showed a gradual decrease between 7 and 9 years. These findings are in agreement with those reported in similar experiments conducted with English-speaking children (Montgomery, 2000, 2002; Montgomery et al., 1990; Montgomery & Leonard, 1998) and confirm that word detection in school-aged children improves with age. Contrary to our expectations, all age groups detected the word targets faster in the Slow speech condition than in both the Prosody and the Sung conditions. A difference in pitch saliency across conditions could not account for the better performance in the Slow speech condition, since differences in semi-tones between the target word and the preceding syllable were strictly identical in the Prosody condition, and these differences were not significantly different from those in the Sung condition. A possible interpretation for the different RTs found in the Slow speech condition is that, in each trial, children were instructed to first listen to a spoken syllable before they had to detect it in a sentence. The words they heard before they had to detect them were thus acoustically more similar than in the other two conditions because the target words were spoken rather than sung. Alternatively, although they were not instructed to do so, it is possible that some children repeated silently or memorized an abstract representation of the word they had to detect. In the latter case, the representation of the monosyllabic word in their inner speech was likely more similar in terms of acoustic features to the word they have to detect in the Slow speech condition than in the other two conditions. Finally, level of exposure could account for the results found in the Slow speech condition. Indeed, children are intensively exposed to speech from birth, most likely less exposed to songs, and even less to the type of speech in the Prosody condition.

Another surprising finding was the absence of a difference between the Prosody and the Sung condition. The melody in the Prosody condition did not respect the rules of Western musical harmony and thus, following the results of Schellenberg et al. (2005) in native French-speaking children, shorter RTs were predicted in the Sung than in the Prosody condition. Note, however, that Schellenberg et al. presented all target words at final positions, and they used only two word targets (/*di*/ and /*du*/) whereas we used 22 different word targets. If melodic expectations apply only to the last position of a musical sequence, this could explain why the detection of words was not facilitated in the Sung condition.

The linguistic context effect was confirmed in that shorter RTs were found in the End than in the Beginning position, irrespective of the condition. This finding is congruent with that of previous work (Montgomery, 2000; Montgomery et al., 1990) and suggests that the linguistic processing of the sentences helped the participants in the detection of target words. However, this position effect could also be the result of the characteristics of our experimental setting. Namely, children were instructed that a target word would be present in each trial, and may, thus, have progressively increased their attention, which would be maximal at the end of the sentence. This greater attention at the end of sentences may have resulted in shorter RTs for target words in the End sentence positions than in the Beginning sentence positions.

To summarize, the analysis of RTs on valid responses indicated that older (9- and 10-years old) children detected target words faster than the younger children (7- and 8-years olds). It also showed that word detection was faster in the spoken condition (i.e., Slow speech condition) than in the sung conditions (i.e., the Sung and Prosody conditions), irrespective of the age of the children. We can speculate that the Slow speech condition was easier because all children have been more exposed to spoken than to sung speech. However, because the duration of the phonemes and syllables was equalized across the three conditions, although less artificial than in the other two conditions, the speech in the Slow speech condition remained unnatural, preventing us from drawing firm conclusions about exposure. A second problem with the paradigm in Experiment 1 was that the criterion for the validity of the responses was based on speed, not on accuracy. Word detections were considered accurate as long as the children’s responses fell within the time window in which the target words occurred. However, this constraint can raise problems in testing children with learning disabilities. Experiment 2 aimed to correct these two flaws.

## EXPERIMENT 2: ACCURACY IN WORD DETECTION

The second experiment had five main objectives: 1) to assess accuracy in word detection rather than speed as in Experiment 1; 2) to compare accuracy in word detection in Sung, Prosody, and Slow speech conditions, as well as in a Natural speech condition—that is, a condition with a faster speech rate; 3) to replicate the linguistic context effect; 4) to reveal a melodic context effect by increasing the number of valid responses; 5) to validate the use of the paradigm to assess word detection in children with SLI aged between 7 and 12 years.

From the results of Experiment 1, we hypothesized that performance would increase with age. With respect to the difference between the Slow speech and Natural speech conditions, two hypotheses can be posited: 1) If word detection relies on intelligibility, which is better in slow speech than in natural speech (e.g., Racette, Bard, & Peretz, 2006), children should perform worse in the Natural speech condition in which syllables have a shorter duration, than in the three other conditions; 2) if the results found in the Slow speech condition are due to language exposure, the best performance should be found in the Natural speech condition. As in Experiment 1, we predicted a better performance for word targets located in the End position due to the linguistic context effect. Moreover, the modifications brought to the experimental paradigm might contribute to also reveal a melodic context effect, namely, a better accuracy in the Sung than in the Prosody condition.

### Method

#### Participants

Sixty-eight children (35 girls and 33 boys) aged 7 to 12 years (*M*_age_ = 9.3 years, *SD* = 1.6 years) participated in this study. For further comparison of results in Experiment 3, the children were subdivided into two groups: the Young group (*n* = 37) comprising children aged 7 to 9 years (*M*_age_ = 8.1 years, 19 girls and 18 boys), and the Old group (*n* = 31) comprising children aged 10 to 12 years (*M*_age_ = 10.6 years, 16 girls and 15 boys). They were recruited in four different schools in Reims and in Lille (France). The selection criteria were the same as in Experiment 1.

#### Stimuli

The stimuli set was identical to the one used in Experiment 1. Twenty-four sentences extracted from children’s songs were used in the same three conditions: Slow speech, Sung, and Prosody, to which we added a fourth condition, the Natural speech condition. In this Natural speech condition, sentences were uttered by the professional singer using his natural way of speaking. Sentences in this fourth condition were not acoustically modified and, thus, included the prosodic variations found in normal speech (Appendix F). The durations of the sentences in the Natural speech condition ranged from 3,910 ms to 9,976 ms, with a mean duration of 5,681 ms (*SD* = 1,215 ms), which was significantly shorter than the sentences in the three other conditions, *t*(46) = 5.6, *p* < .001. The syllable duration variability (mean standard deviation of syllables) in the Natural speech condition was also significantly greater than in the other three conditions (117 ms and 90 ms, respectively), *t*(46) = 4.2, *p* < .001. Target and foil syllables were identical to those used in Experiment 1.

#### Procedure

The procedure was identical to Experiment 1 except that in each trial, the child first heard a word that could either be present or absent in the sentence. The stimulus sentence was presented 1,500 ms after hearing the target word, and a picture of a red and a green smiley face appeared on the screen 500 ms after the end of the sentence. The child was instructed to wait until the sentence and smiley had ended before indicating whether the word had been heard by pressing the corresponding key (green for “yes” and red for “no”). The next trial did not begin until the participant responded. Responses were recorded with E-prime 2 software (Psychology Software Tools). Each testing session began with three practice trials.

In order to shorten the duration of the task and to avoid learning effects, each child was tested in the four conditions but in two sessions separated by one week. Words that were present in sentences in one condition were not present in the other one. Sentences were divided into three blocks each followed by a few minutes pause, the duration of which was determined by the participant. The approximate duration of testing was 15 min per session.

### Results

In order to allow the comparison with results of Experiment 1, and to keep only the children who performed above chance level, we calculated individual percentages of correct responses (CR). Across the four conditions, the mean percentage of CRs was 88% (*SD* = 9.6%). Five of the children had a percentage of CRs lower than 70%. After excluding them, the mean percentage of CRs was 90% (*SD* = 6.4). In the subsequent analyses of variance, the percentage of Hits minus False Alarms (FAs), an unbiased accuracy score, was used as the dependent variable.

**Figure 3.**
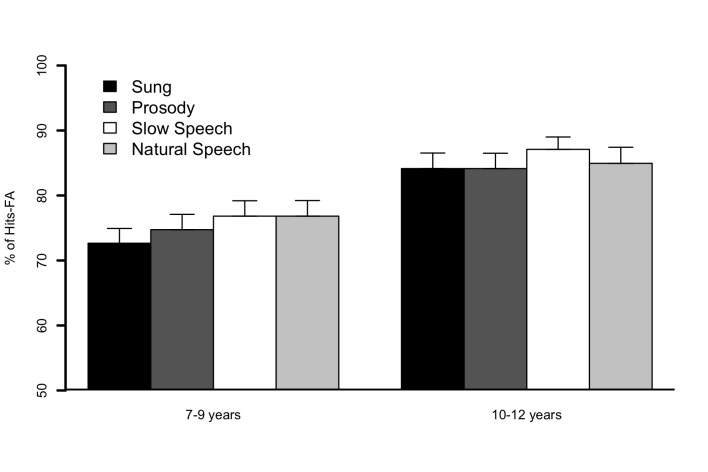
Percentages of Hits − False alarms (FA) in Sung (black bars), Prosody (dark grey bars), Slow speech (white bars), and Normal speech (light grey bars) conditions for children in “7–9 years” and “10–12 years” subgroups of Experiment 2. Error bars represent standard errors of the mean.

**Figure 4.**
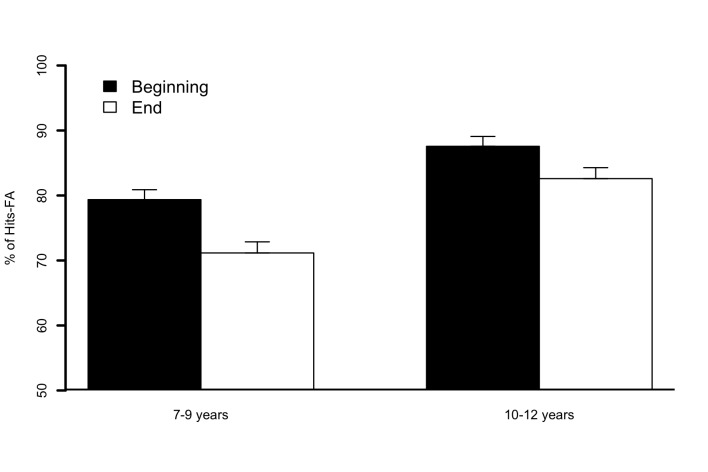
Percentages of Hits – False alarms (FA) for targets that occurred at the beginning (black bars) and end (white bars) positions for children in the “7–9 years” and “10–12 years” subgroups of Experiment 2. Error bars represent standard errors of the mean.

An ANOVA with one between-subjects factor, Age, with two levels (7-9 years, 10-12 years), and two within-subject factors, Condition, with four levels (Slow speech, Sung, Prosody, Natural speech), and Target Position, with two levels (Beginning, End) was run on the percentage of Hits minus FAs. The results, illustrated in Figure 3, showed a main effect for Age, *F*(1, 61) = 10.7, *p* < .002, whereby the 10-12 year group obtained higher scores (*M* = 85.1) than the 7-9 year group (*M* = 75.3). There was no effect of condition, *F*(3, 183) = 1.05, *ns*. An effect of Position, illustrated in Figure 4, was also obtained, *F*(1, 61) = 29.5, *p* < .001), this score being higher when the target was located at the beginning (*M* = 83.4) than at the end (*M* = 76.8). Finally, none of the interactions were significant, Age × Condition, *F*(3, 183) = 0.2, *ns*; Age × Position: *F*(1, 61) = 1.8, *ns*; Condition × Position, *F*(3, 183) = 0.6, *ns*; Age × Condition × Position, *F*(3,183) = 2.2, *ns*.

### Discussion

The percentage of CRs in this second experiment was higher (90%) than that obtained in the first experiment (49.5%) and comparable to that reported in previous studies by Montgomery and collaborators (Montgomery, 2000; Montgomery et al., 1990; Montgomery & Leonard, 1998) who reported mean percentages of CRs ranging from 88% to 98%.

As predicted, the percentage of Hits minus FAs increased with age, which confirmed a progressive improvement in word detection accuracy. The abilities in word detection, thus, appeared to improve until pre-adolescence. Contrary to our predictions and to results from Experiment 1, no effect of condition was found. Accuracy in word detection was, thus, not influenced by the duration of the syllables since accuracy in the Natural speech condition did not differ from that in the three other conditions, all of which included syllables with longer durations.

Contrary to both the findings in Experiment 1, and the predictions based on the linguistic context effect, we found a position effect in the opposite direction: Children were more accurate for word targets in the Beginning than in the End positions. This apparent discrepancy may stem from procedural differences between the two tasks. In Experiment 1, the children were requested to press a button as quickly as possible when they detected the word. In Experiment 2, the children had to decide whether or not the target word was present in the sentence by pressing the Yes or the No button, without any time constraint. Both requirements and expectations, thus, differed in the two tasks with respect to the position of the target within the sentence. In Experiment 1, expectations were certainly higher at the end than at the beginning of the sentence because children were told that each sentence contained a target, and that their task was to detect it as quickly as possible. Thus, it is possible that a higher level of expectation led to shorter RTs. In Experiment 2, the detection of target words located at the end of sentences may have required more sustained attention to keep the target in short-term memory compared to the detection of targets located at the beginning of sentences. Thus, the task may have been less demanding when the target was located at the beginning rather than at the end of sentences.

To summarize, analysis of word detection accuracy indicated that older children aged 10 to 12 years detected more words than younger children aged 7 to 9 years, whether the sentences were spoken (slow speech or natural speech) or sung (in the original melodic score or in a nonmelodic score extracted from speech prosody). Both young and older children detected words better at the beginning than at the end of sentences.

An important objective of Experiment 2 was to modify the paradigm so as to increase the percentage of valid responses. We have achieved this goal in that the overall percentage of CRs was significantly higher in Experiment 2 (90%) than in Experiment 1 (49.5%). The findings in Experiment 2 showed that children from 7 to 12 years were able to perform the task, and that the modified paradigm is suitable for testing in children with language deficits.

## EXPERIMENT 3: WORD DETECTION IN CHILDREN WITH SLI

Montgomery (2000, 2002; Montgomery & Leonard, 1998) reported that children with SLI showed impairments in the detection of words in sentence stimuli spoken at a normal rate of speech. The objective of Experiment 3 was to investigate whether children with SLI would detect words with a longer duration better than words spoken at a normal rate of speech. Additionally, we wished to investigate whether there was an impact of melodic context on word detection.

A significant age effect was found in both Experiments 1 and 2, revealing that older children detected words more quickly and accurately than younger children. Abilities in word detection, thus, appear to improve with language development. Assuming that the development of language abilities in children with SLI is delayed (Hoff-Ginsberg, 2005; Saint-Pierre, 2006; Saint-Pierre & Béland, 2010), we also predicted an age effect within this population. More specifically, older children with SLI (10 to 12 years) should perform inferior to age-matched children with TLD but not different to younger children (7 to 9 years) with TLD. In addition, the performance of young children with SLI (7 to 9 years) should be inferior to that of age matched children with TLD. Results of Experiment 2 showed no effect of condition on word detection accuracy for children with TLD. As we were using the same paradigm, no condition effect was predicted for children with TLD in Experiment 3. As for the population of children with SLI, previous studies have used normal speech rate only, precluding specific hypotheses on the effect of condition. We could nonetheless predict that words with longer syllable durations (sung or slow speech) would be more easily detected than words spoken at a normal rate of speech. Finally, we predicted to also find a position effect in both children with TLD and children with SLI, with better accuracy on targets located at the beginning than at the end of sentences.

### Method

#### Participants

We recruited 16 children (10 boys and 6 girls, *M*_age_ = 10.1 years) with Specific Language Impairment (SLI group) in schools with special programs for children with language disorders in Reims and Charleville Mézičres (France). As in Experiment 2, the 16 children were divided into two groups: the Young group, composed of the 9 children aged 7 to 9 years (*M*_age_ = 8.9 years, 4 girls and 5 boys), and the Old group, composed of the 7 children aged 10 to 12 years (*M*_age_ = 11.6, 2 girls and 5 boys). All children had been enrolled in speech therapy training for 2 to 10 years (*M*_year_ = 5.8). They suffered from deficits affecting either expressive or receptive language, or both, with different levels of severity (see Table 2). The details of the tests are presented in Appendix E.

**Table 2.** Experiment 3. Language Data for Children in SLI Group

SLI Group														TLD Group			
SLI Subjects (code)	Speech Therapy-Number of Years of Rehabilitation	Gender	Age	Non Verbal Intelligence (percentile)	Language Tests									TLD Subjects (code)	Gender	Age	Non Verbal Intelligence (percentile)
					Receptive Language		Expressive Language			Metaphonology							
					Lexical Comprehension(months)	Syntactical Comprehension	Phonological Production	Lexical Production	Syntactical Production	Initial Phoneme Elison	Phoneme Reversal	Initial Phoneme Addition	Final Phoneme Elison				
1	6	M	9,96	53	-	-	< –2	-	-	-*	-*	–1/–2	-*	A	M	10,28	50
2	5	M	10,77	91	-	-	< –2	-	< –2	-*	–1/–2	-*	< –2*	B	M	10,27	75
3	3	F	9,03	57	–18/–24	–1/–2	< –2	–1/–2	< –2	< –2*	< –2*	< –2*	< –2*	C	F	8,65	25
4	7	M	9,87	39	< –24	-	< –2	-	< –2	< –2*	< –2*	< –2*	< –2*	D	M	9,25	10
5	7	M	9,18	25	–18/–24	< –2	< –2	–1/–2	< –2	< –2*	< –2*	< –2*	< –2*	E	M	9,56	50
6	4	F	8,42	68	< –24	-	< –2	–1/–2	< –2	< –2	< –2	< –2	< –2	F	F	8,66	25
7	5	F	8,93	16	-	–1/–2	< –2	–1/–2	< –2	< –2*	< –2*	< –2*	< –2*	G	F	9,11	25
8	2	M	7,35	53	< –24	< –2	< –2	< –2	< –2	< –2	< –2	< –2	< –2	H	M	7,66	50
9	6	F	8,85	25	< –24	< –2	< –2	< –2	–1/–2	< –2*	< –2*	< –2*	< –2*	I	F	8,37	25
10	10	M	12,53	25		-	< –2	-	-	< –2*	< –2*	–1/–2*	–1/–2*	J	M	12,66	75
11	6	M	12,14	34	< –24	-	< –2	< –2	-	US	US	US	US	K	M	12,34	75
12	5	M	8,73	10	< –24	< –2	< –2	–1/–2	–1/–2	< –2*	< –2*	< –2*	< –2*	L	M	8,19	10
13	4	F	10,38	50	< –24	-	< –2	-	–1/–2	< –2*	< –2*	< –2*	< –2*	M	F	10,05	50
14	9	F	12,87	75	< –24	–1/–2	< –2	-	< –2	< –2*	< –2*	< –2*	< –2*	N	F	12,90	10
15	7	M	11,30	50	-	-	< –2	-	-	-*	-*	–1/–2	-*	O	M	11,79	95
16	7	M	11,26	9	-	-	< –2	-	–1/–2	-*	-*	-*	-*	P	M	11,67	50

*Note*. Scores below 2 *SD* (< −2) or between 1 and 2 *SD* (−1/ −2) from the normal control scores, scores 24 months (<−24 months), or between 18 and 24 months (−18/ −24 months) below the expected level, normal scores (−) and unavailable scores (US). * Values representing scores compared to the children norms for 8.5-years-old (norms being unavailable for older children).

Sixteen healthy children with TLD, aged 7 to 12 years (6 girls and 10 boys, *M*_age_ = 10.1 years), were paired, as much as was possible, to children with SLI for age, sex, and scores in non-verbal intelligence tests (see description of the tests in Appendix E). Similarly to the SLI group, the TLD group was divided into two groups: the Young group composed of the 8 children aged 7 to 9 years (*M*_age_ = 8.9 years, 4 girls and 4 boys), and the Old group, composed of the 8 children aged 10 to 12 years (*M*_age_ = 11.6; 2 girls and 6 boys). None of the children in the TLD group had a history of language disorder, as reported by their parents.

As reported in Table 2, non-verbal intelligence expressed in percentile was within the average range (> 9th percentile) for participants in both groups. The SLI and TLD groups did not differ either in age, *t*(30) = 0.01, *ns*, or non-verbal intelligence, *t*(30) = 0.4, *ns*. No children in either group had received musical training, and had no reported auditory, physiological, or neurological problems. All participants received a short hearing screen using an audiometer. Sounds were presented to the left and the right ear at a range of frequencies (125, 250, 500, 1000, 2,000, 4,000 Hz), and all participants were sensitive to sounds within the 20 dB HL range.

#### Stimuli and procedure

For the word detection task, the stimuli and procedure were similar to Experiment 2.

### Results

A repeated measures ANOVA was run using the percentage of Hits minus that of FAs as the dependent variable. The ANOVA had two between-subjects factors, Group with two levels (SLI group, TLD group), and Age with two levels (7-9 years, 10-12 years), and two within-subject factors, Conditions with four levels (Slow Speech, Natural speech, Sung, Prosody), and Target Position with two levels (Beginning, End).

**Figure 5.**
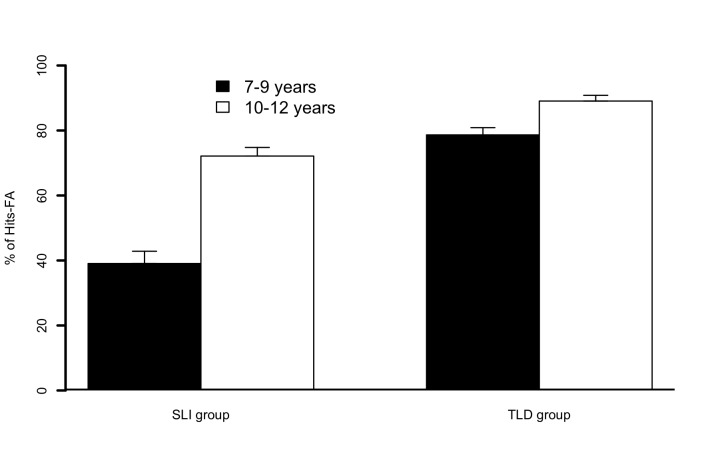
Percentages of Hits − False alarms (FA) in specific language impairment (SLI) and typical language development (TLD) groups for children in the “7–9 years” (black bars) and “10–12 years” (white bars) subgroups of Experiment 3. Error bars represent standard errors of the mean.

**Figure 6.**
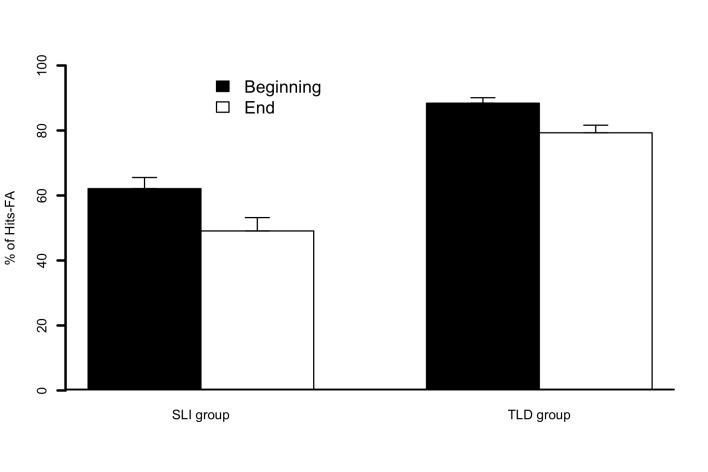
Percentages of Hits − False alarms (FA) for children in specific language impairment (SLI) and typical language development (TLD) groups for targets that occurred at the beginning (black bars) and end (white bars) positions of Experiment 3. Error bars represent standard errors of the mean.

The results revealed a Group by Age interaction, *F*(1, 28) = 6.1, *p* < .02 (Figure 5). Post-hoc comparisons with Holm-Bonferroni corrections showed that older children in the SLI group obtained higher Hits FA percentages (*M* = 72.1) than younger children in the SLI group (*M* = 39.1), whereas this was not the case for children in the TLD groups (*M* for older children = 89.1; *M* for younger children = 78.6). Furthermore, post-hoc comparisons with Holm-Bonferroni corrections did not reveal a significant difference between Hits FA percentages for older children in the SLI group and younger children in the TLD group. The analysis showed an effect of Group, *F*(1, 28) = 38.1, *p* < .001), whereby children in the SLI group (M% Hits FAs = 56.6) were impaired compared to children in the TLD group (*M*_% Hits FAs_ = 83.8). There was also an effect of Age, *F*(1, 28) = 22.5, *p* < .001, whereby the 7-9 year group (*M*_% Hits FAs_ = 58.9) obtained lower scores than the 10-12 year group (*M*_% Hits FAs_ = 80.6). As illustrated in Figure 6, the ANOVA also revealed an effect of Position, *F*(1, 28) = 22.9, *p* < .001, whereby the percentage of Hits FAs was higher when the target was located at the Beginning position (*M*_% Hits FAs_ = 75.3) than at the End position (*M*_% Hits FAs_ = 64.2). However, the effect of Condition was not significant, *F*(3, 84) = 0.1, *p* > .05, nor were any of the other interactions (all non-significant ps > .05), Group × Condition, *F*(3, 84) = 1.3; Age × Condition, *F*(3, 84) = 0.8; Group × Position, *F*(1, 28) = 0.7; Age × Position, *F*(1, 28) = 2.3; Condition × Position, *F*(3, 84) = 0.3; Group × Age × Condition, *F*(3, 84) = 2.5; Group × Age × Position, *F*(1, 28) = 0.9; Group × Condition × Position, *F*(3, 84) = 0.3; Age × Condition × Position, *F*(3, 84) = 0.8; Group × Age × Condition × Position, *F*(3, 84) = 1.1.

In the SLI group, scores in word detection in the four conditions (Slow speech, Natural speech, Sung and Prosody conditions) were positively and significantly correlated with scores in metaphonological tasks: Slow speech, *r*(13) = 0.51, *p* = .03; Natural speech, *r*(13) = 0.52, *p* = .02); Prosody, *r*(13) = 0.47, *p* = .04; Sung conditions, *r*(13) = 0.80, *p* < .001.

### Discussion

As predicted, children in the SLI group scored lower than children with TLD, which confirms findings of previous studies in English-speaking children (Montgomery, 2000, 2002; Montgomery & Leonard, 1998). The ANOVA also revealed higher scores with increasing age, but only in children with SLI. As predicted, the scores of older children with SLI were close to the scores of the younger children with TLD, which supports findings of a two-year delay in language development. The absence of an age effect in the children with TLD, contrary to findings in Experiment 2, may be accounted for by the smaller group size in Experiment 3. The fact that mean scores did not differ from Experiments 2 to 3, neither for the young (Experiment 2 = 75.3%; Experiment 3 = 78.6%) nor the old (Experiment 2 = 85.1%; Experiment 3 = 89.1%) subgroups of children with TLD, supports the interpretation of a masked age effect in Experiment 3. The size of the group evidently did not influence the emergence of a group effect in children with SLI to the same extent because the mean score of young children with SLI was lower (39.1%) than that of young children with TLD in both Experiments 2 (75.3 %) and 3 (78.6%). This was not the case for the older children with SLI who obtained scores close to those of the young children with TLD in both Experiments.

The position effect found in Experiment 2 was replicated in Experiment 3, in both children with TLD and children with SLI, without an interaction with age. Consistent with our interpretation of Experiment 2’s results, this robust effect is considered to reflect attentional abilities.

To summarize, the two subgroups of children with SLI showed impairments in word detection compared to children with TLD, although groups were matched for sex, chronological age, and non-verbal IQ scores. There was no effect of condition, however. Even the group of young children with SLI, who obtained the lowest scores, did not benefit from words with a longer duration (Slow speech condition) or words that were sung (Sung and Prosody conditions) compared with words spoken at a natural rate of speech (Natural speech condition). Results of both Experiments 2 and 3 revealed that word detection abilities seem to show a progressive development and are not yet at ceiling levels at 12 years of age. Findings from Experiment 3 indicate that the development of these abilities in children with SLI is delayed, and these abilities continue to develop with an even steeper slope.

## GENERAL DISCUSSION

This study aimed to assess the development of word detection abilities in children aged 7 to 12 years with TLD and SLI. Based on the facilitation effect documented in the literature, we hypothesized that word detection would be easier in sung than in spoken sentences. In order to single out one acoustic difference between spoken and sung sentences, we created conditions in which the sung and the spoken sentences were equated for syllable durations and pitch variations. The sole difference between the two sentence types (Sung and Slow speech conditions) was that pitch variations in the sung sentences followed the harmony rules of Western music, whereas those in the spoken sentences did not.

We failed to find any advantage of sung over spoken sentences, either in the group of children with TLD, or the group of children with SLI. Moreover, no facilitation effect was found when we compared the conditions located at the two extremities of the continuum from spoken to sung sentences—that is, sentences spoken at a natural rate of speech (characterized by short syllable durations and large pitch variations) to sung sentences (characterized by long syllable durations and small pitch variations). These findings contrast with the documented advantage of sung over spoken speech in verbal learning in children (Lebedeva & Kuhl, 2010; Thiessen & Saffran, 2009) and in adults (Schön et al., 2008). Given that the present study and Schön et al.’s study were carried out in French, we cannot attribute the discrepancy between the results to differences between syllable-timed language (i.e., French) and stress-timed language (i.e., English). This null effect cannot be attributed to a ceiling or a floor effect either because the performance improved with age in all three groups of children with TLD, as well as in the group of children with SLI. Both the TLD and SLI groups also consistently showed a word position effect. We interpreted this effect to be a strong indication that both groups understood the task and performed it in a similar way. Age was also found to have a consistent effect in both the TLD group and SLI groups, which indicates that word detection abilities slowly progressed from 7 to 12 years. The scores of the older children in the SLI group were at the level of the scores of the younger children in the TLD group. Moreover, their scores in word detection were correlated with their scores in metaphonological tasks. These findings are consistent with the phonological delay hypothesis in SLI (Hoff-Ginsberg, 2005; Saint-Pierre, 2006), which has also been reported in reading impairment (Saint-Pierre & Béland, 2010).

Another interesting consideration concerns the influence of rhythm. Our primary focus was to determine the effect of pitch and melody on word detection, and, as such, we specifically isolated and manipulated this pitch dimension. While this method is certainly not without merit and it is important to understand the relative contributions of different musical dimensions, our results may be explained by the fact that syllables were produced in an isochronous manner across Sung, Prosody, and Slow speech conditions. In this case, it seems likely that syllables in the same sentence (over these three conditions) had more similar syllable durations, and may, thus, have contributed to the results. The fact that syllable duration was indeed significantly more variable in the Natural speech condition compared to the other three conditions supports the idea that variability of syllable durations may contribute to speech processing. Rhythm influences pitch processing in both adults (Jones, Moynihan, MacKenzie, & Puente, 2002) and infants (Hannon & Johnson, 2005), and may contribute to the hierarchical understanding of musical pitch (Järvinen & Toiviainen, 2000; Palmer & Pfordresher, 2003). Thus, it may be the case that melodic context alone in the Sung and Prosody conditions was not sufficient to impact on word detection, and temporal cues may have strengthened pitch cues. Also concerning this issue, while one might intuitively presume that syllabic isochrony may facilitate speech comprehension (as has been shown for production in speech disfluent populations, Packman, Onslow, & Menzies, 2000; Thaut, 2005), it may be the case that syllable isochrony masks important durational cues required for efficient word detection. Metrical structures in speech—the alternation of prominences, afforded in part by syllabic durational differences—provide important cues for speech segmentation and contribute to speech processing in infants (Johnson & Jusczyk, 2001; Morgan & Saffran, 1995) and adults (Pitt & Samuel, 1990; Roncaglia-Denissen, Schmidt-Kassow, & Kotz, 2013; Rothermich, Schmidt-Kassow, & Kotz, 2012). The beneficial effect of musical metrical cues on sentence perception has also been shown in native French-speaking children with SLI, dyslexia (Przybylski et al., 2013), and hearing impairment (Cason, Hidalgo, Isoard, Roman, & Schön, 2015). We can, therefore, propose that the presence of durational segmentation cues in the Sung and Prosody conditions might have resulted in greater word detection abilities.

We conclude that children with SLI were impaired in comparison to children with TLD. In particular, we note that their word detection skills were significantly correlated with their performance on tests of metaphonological awareness. Overall, we found no effect of the isolated musical dimension—pitch variations—on language processing, but future studies may investigate the effect of other musical dimensions. Further, if music and language do share common neural substrates and common auditory working memory (Christiner & Reiterer, 2013), one would expect that musical abilities, at least those abilities that are required to process sung sentences, would be impaired in children with SLI. Namely, the absence of a benefit of pitch in word processing may be due to an impaired musical processing in children with SLI. This also raises questions about the pitch processing abilities of these children, although these results could also reflect the sensitivity of our paradigm. Further investigations by our team, thus, aim to document the development of musical abilities in children with SLI.

## Figures and Tables

**Figure B1.**
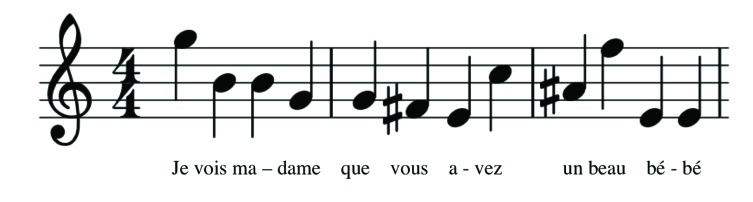
Pitch variations in the Slow speech and Prosody conditions.

**Figure B2.**
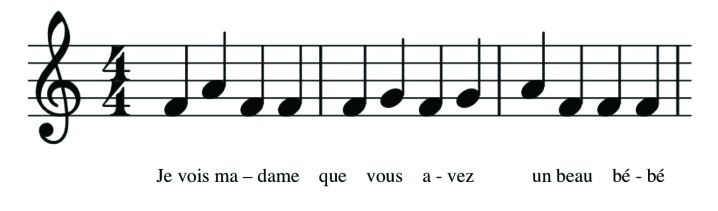
Pitch variations in the Sung condition.

**Figure F.**
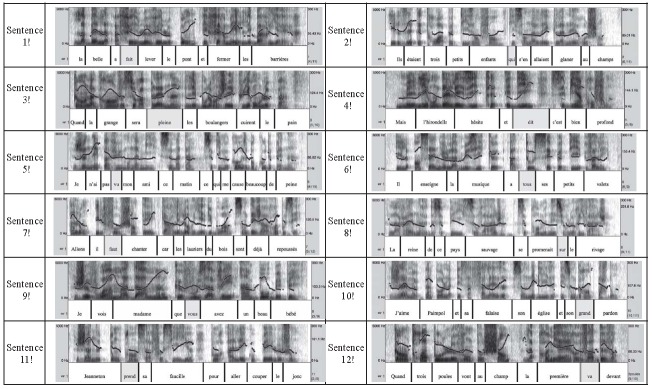
Prosodic structures of sentences

## Autor’s notes

The project was approved supported by grants from SSHRC 410-2010-1492 to Renée Béland and from l’Agence Nationale de la Recherche (ANR-09-BLAN-0310-02) and l’Institut Universitaire de France to Séverine Samson.

## Appendix A

**Table A.** Song Names, Lyrics, and Target Words Used in the Three Experiments

	Song Names	Sentence Number	Syllabic Structure of Target	Sentences and Targets	
				First Half of the Sentence	Second Half of the Sentence
Targets in the First Half of the Sentence	Le chateau	1	CV	La belle a ***fait*** lever le	pont et fermer les barričres
	Les patrons	3	CCVC	Quand la grange sera ***pleine***	les boulangers cuiront le pain
	Bonjour Mademoiselle	5	CV	Je n'ai pas ***vu*** mon ami ce matin	ce qui me cause beaucoup de peine
	Nous n'iront plus au bois	7	CV	Allons il ***faut*** chanter car les lau	-riers du bois sont déjŕ repoussés
	Le beau bébé	9	CV	Je vois madame que ***vous***	avez un beau bébé
	Jeanneton prend sa faucille	11	CCV	Jeanneton ***prend*** sa faucille	pour aller couper le jonc
	Le roi a fait battre tambour	13	CVC	align="right"Marquis ne te fâche ***donc***	pas t'auras ta récompense
	Arlequin	15	CV	Il vend des ***bouts*** de réglisse	meilleurs que votre bâton
	Monsieur De La Palisse	17	CVV	Mais il ne manqua de ***rien***	dčs qu'il fut dans l'abondance
	Jeannot chasseur	19	CVC	Dans un pré ***vert*** comme l'oseille	Maître Jeannot dresse l'oreille
	Les éléphants	21	CV	Ils se roulent dans la ***boue***	les feuilles et la poussičre
	Arlequin	23	CVV	Arlequin ***tient*** sa boutique	dessous un grand parasol
Targets in the Second Half of the Sentence	La legende de Saint Nicoles	2	CV	Il étaient trois petits enfants	***qui*** s'en allaient glaner aux champs
	C'était sur la tourelle	4	CV	Mais l'hirondelle hésite	et ***dit*** c'est bien profond
	Arlequin	6	CV	Il enseigne la musique	ŕ ***tous*** ses petits valets
	L'orpheoniste	8	CVC	La reine de ce pays sauvage	se promenait ***sur*** le rivage
	La paimpolaise	10	CCV	J'aime Paimpol et sa falaise	son église et son ***grand*** pardon
	Quand trois poules	12	CV	Quand trois poules vont au champ	la premičre ***va*** devant
	La bonne aventure ô gué	14	CV	Je suis un petit poupon de belle figure	***qui*** aime bien les bonbons et les confitures
	Oh Ninette	16	CV	Les renards et les vautours	s'enfuiront de ***nos*** contrées
	Dame Tartine	18	CV	Elle épousa monsieur Gimblette	coiffé d'un ***beau*** fromage blanc
	Le petit bossu	20	CV	Quand le p'tit bossu va chercher de l'eau	il n'y va jamais ***sans*** son petit seau
	Farandole provençale	22	CCV	Que votre danse ait la cadence	des ***frais*** rameaux que la brise balance
	Le petit homme	24	CVC	Il en reste un wagon	pour lui ***faire*** le capuchon

*Note*. Target words in bold italics.

## Appendix B

Example of pitch variation in the three conditions.

## Appendix C

**Table C.** Repartition of the Sentences Divided in Three Blocks of 16 Sentences

Sentence Number	Block 1	Block 2	Block 3
1-4	×	×	
5-8	×		×
9-12	×	×	
13-16		×	×
17-20	×		×
21-24		×	×

## Appendix D

**Table D.** Repartition of Blocks in Six Combinations

Combinations	Sung Condition	Slow speech Condition	Prosody Condition
A	block 1	block 2	block 3
B	block 1	block 3	block 2
C	block 2	block 1	block 3
D	block 2	block 3	block 1
E	block 3	block 1	block 2
F	block 3	block 2	block 1

## Appendix E

**Table E.** Language and Non-Verbal Intelligence Tests Used in Children With SLI

Cognitive Functions		Tests
Receptive Language	Lexical Comprehension	EVIP (Dunn, Thériault-Whalen, & Dunn, 1993) – French adaptation of PPVT-revised
		or
		Subtest « désignation », TVAP (Deltour & Hupkens, 1980) - « Pointing » subtest, Active and Passive Vocabulary Test
	Syntactic Comprehension	ECOSSE (Lecocq, 1996) - French version of TROG
ExpressiveLanguage	Phonological Production	Subtest « Phonologie », L2MA (Chevrie-Muller, Simon, & Fournier, 1997) - « Phonology » subtest, Batteryfor child cognitive assessment
		or
		Subtest « Phonologie », N-EEL (Chevrie-Muller & Plaza, 2001) – « Phonology » subtest, « New tests for language examination »
	Lexical Production	Subtest « Dénomination », L2MA (Chevrie-Muler et al., 1997) - « Picture naming » subtest, L2MA
		or
		Subtest « Vocabulaire », N-EEL (Chevrie-Muller & Plaza, 2001) - « Vocabulary » subtest, N-EEL
	Syntactical Production	Subtest « Intégration morphosyntaxique », L2MA (Chevrie-Muler et al., 1997) - « Morphosyntax production subtest, L2MA
		or
		TCG-R (Deltour, 1998) – Revised grammar cloze test
	Metaphonology	Subtests « Conscience phonologique », N-EEL (Chevrie-Muller & Plaza, 2001) - « Phonological awareness» tests, N-EEL
Non-Verbal Intelligence		The Performance subtests of the WISC-III (Wechsler, 1996)
		or
		The Perceptual Reasoning Index of the WISC-IV (Wechsler, 2005)
		or
		The Colored Progressive Matrices test (Raven, 1998)

*Note*. EVIP = Échelle de Vocabulaire en Images de Peabody; PPVT = Peabody Picture Vocabulary Test; TVAP = Test de Vocabulaire Actif et Passif; ECOSSE = Épreuve de COmpréhension Syntaxico-Sémantique; TROG = Test for Reception of Grammar; L2MA = batterie « Langage oral, Langage écrit, Mémoire, Attention »; N-EEL = Nouvelles Épreuves pour l’Examen du Langage; WISC-III = Wechsler Intelligence Scale for Children - third edition; WISC-IV = Wechsler Intelligence Scale for Children - fourth edition
